# Evaluating the anti-neuropathic effects of the thymol-loaded nanofibrous scaffold in a rat model of spinal cord injury

**DOI:** 10.3389/fphar.2025.1507397

**Published:** 2025-04-04

**Authors:** Roshanak Amirian, Pardis Mohammadi Pour, Hassan Maleki, Sajad Fakhri, Sedigheh Asgary, Mohammad Hossein Farzaei, Javier Echeverría

**Affiliations:** ^1^ Pharmaceutical Sciences Research Center, Health Institute, Kermanshah University of Medical Sciences, Kermanshah, Iran; ^2^ Student Research Committee, School of Pharmacy, Kermanshah University of Medical Sciences, Kermanshah, Iran; ^3^ USERN Office, Kermanshah University of Medical Sciences, Kermanshah, Iran; ^4^ Department of Pharmacognosy, Faculty of Pharmacy, Tehran University of Medical Sciences, Tehran, Iran; ^5^ Isfahan Cardiovascular Research Center, Cardiovascular Research Institute, Isfahan University of Medical Sciences, Isfahan, Iran; ^6^ Departamento de Ciencias del Ambiente, Facultad de Química y Biología, Universidad de Santiago de Chile, Santiago, Chile

**Keywords:** spinal cord injury, motor activity, neuropathic pain, thymol-scaffold, chitosan, poly(vinyl alcohol)

## Abstract

**Background:**

Spinal cord injury (SCI) is a debilitating condition characterized by partial or complete loss of motor and sensory function caused by mechanical trauma to the spinal cord. Novel therapeutic approaches are continuously explored to enhance spinal cord regeneration and functional recovery.

**Purpose:**

In this study, we investigated the efficacy of the poly(vinyl alcohol) and chitosan (PVA/CS) scaffold loaded with different thymol concentrations (5, 10, and 15 wt%) in a rat compression model for SCI treatment compare to other (e.g., thymol and scaffold) control groups.

**Results and discussion:**

The thymol-loaded scaffold exhibited a smooth surface and a three-dimensional nanofibrous structure with nanoscale diameter. The conducted analyses verified the successful incorporation of thymol into the scaffold and its high water absorption, porosity, and wettability attributes. Behavioral assessment of functional recovery showed improving sensory and locomotor impairment. Furthermore, histopathological examinations indicated the regenerative potential of the thymol-loaded nanofiber scaffold, by neuronal survival.

**Conclusion:**

Therefore, these findings suggest that the thymol-loaded nanofibrous scaffolds have promising pharmacological activities for alleviating neuropathic pain and addressing complications induced by SCI.

## 1 Introduction

Neuropathic pain is a sort of continual ache that occurs when damage or disorder affects the somatosensory nervous system, impacting 7%–10% of the world population (Colloca et al., 2017). Neuropathic pain due to spinal cord injury (SCI), can bring about a lack of sensation, movement, bowel and bladder management, and can range in rigor from mild to intense (Alizadeh et al., 2019; Joosten and Franken, 2020). Recent investigations have discerned that the modulation of apoptotic gene expression, with potential implications for neuropathic pain, is orchestrated by reactive oxygen species (ROS) and inflammatory factors (Siniscalco et al., 2007). Consequently, ROS assumes a pivotal role in the cascade of events associated with nerve damage, concomitant with processes such as caspase activation, glial activation, and mitochondrial dysfunction (Andersen, 2004). A spectrum of pain medicinal drugs, such as anticonvulsant pills, can also correctly lessen neuropathic pain. Gabapentin is the first line of medication in neuropathic pain (Levendoğlu et al., 2004), and anti-inflammatory drugs such as nonsteroidal anti-inflammatory drugs (NSAIDs) (Colloca et al., 2017; Hayta and Elden, 2018; Terzi et al., 2018; Gurba and Haroutounian, 2022), are effective in reducing neuropathic pain and may be used in combination with other pain medication. Topical treatments containing capsaicin a compound in chili peppers, acts on the transient receptor potential cation channel subfamily V member 1 (TRPV1) receptor to provide analgesic effects by desensitizing pain pathways (Hall et al., 2020; Liau et al., 2020; Zheng et al., 2020; Olusanya et al., 2023).

Herbal medications are sometimes used as complementary or alternative treatments for SCI (Nayak et al., 2001; Boyd et al., 2019). While their therapeutic efficacy has yet to undergo extensive scientific investigation, herbal remedies are garnering increasing attention as a potentially viable alternative owing to their favorable safety profiles in contrast to conventional pharmacological agents. Prominent herbal constituents employed in the context of SCI management encompass curcumin (Indermun et al., 2019; Jin et al., 2021; Li et al., 2021) and ginger (Andrei et al., 2022; Shen C.-L. et al., 2022). Renowned for their anti-inflammatory attributes, these herbs hold promise in mitigating the pain and inflammation typically associated with spinal cord injuries. Furthermore, noteworthy research has indicated that *Ginkgo biloba* L. [Ginkgoaceae] (Kim et al., 2009; Al-Adwani et al., 2019) may augment cerebral and spinal cord blood circulation, accompanied by potential neuroprotective properties that could facilitate the recuperative process following spinal cord injuries. Additionally, thymol (2-isopropyl-5-methylphenol) is the main naturally occurring monoterpene phenol prevalent in thyme essential oils, particularly *Thymus vulgaris* L. [Lamiaceae], and has demonstrated anti-inflammatory and analgesic qualities (Costa et al., 2019; Sheorain et al., 2019; Wang W. et al., 2020; Zheng et al., 2022). This effective agent is appropriate to utilize as an anti-inflammatory activity by inactivating the calcium channels or initiating a corresponding decrease in elastase (Braga et al., 2006), and antioxidant activity was proven by the (2,2-diphenyl-1-picrylhydrazyl) (DPPH) and human red blood cell stabilization methods (Sheorain et al., 2019). However, thymol has a strong irritating/bitter taste and a low aqueous solubility. These physicochemical characteristics lead to the development of pharmaceutical preparation that covers these undesirable characteristics (Nieddu et al., 2014). Besides herbs, the use of scaffolds and thymol in SCI treatment is an area of abiding research in regenerative medicine (Qian et al., 2021; Zheng et al., 2022).

Scaffolds are critical in SCI management, as they provide structural support that closely mimics the natural extracellular matrix (ECM) and thus present a conducive environment for cell adhesion, proliferation, and differentiation, which are key to tissue regeneration (Stokols and Tuszynski, 2004). Nanofibrous scaffolds, often fabricated by the electrospinning process, possess particular advantages due to their nanoscale fibrous architecture, which closely mimics the composition of the ECM. This resemblance increases the scaffold’s ability for cell adhesion, migration, and nutrient exchange, which are essential in nerve tissue regeneration (Zhang et al., 2019).

Nanofibrous scaffolds are also effective platforms for localized drug delivery, allowing controlled, sustained release of therapeutic agents directly to the injury site (Faccendini et al., 2017). This localized approach is advantageous for SCI treatment, as it delivers a concentrated dose of bioactive compounds like thymol precisely where needed, reducing systemic side effects (Faccendini et al., 2017).

By combining poly (vinyl alcohol (PVA) and chitosan (CS), two polymers favorable for the fabrication of electrospun nanofibers (NFs), we strengthen the scaffold and make it more stable ensuring it can withstand compression without losing its shape or structural integrity. This is crucial, for creating an environment that promotes tissue regeneration and aids in the recovery of cord function. Additionally, the hydrophilic properties of PVA and CS contribute to the scaffold’s functionality. The scaffold’s porosity and permeability are vital for infiltration and nutrient exchange. By processing PVA and CS create a scaffold with high porosity allowing for the diffusion of oxygen, nutrients, and waste products. Furthermore, such nanofibrous scaffolds can effectively encapsulate and deliver substances.

In this study, we loaded the nanofibrous scaffold with thymol, a compound with well-reported analgesic anti-inflammatory and antioxidant properties (Yanishlieva et al., 1999; Braga et al., 2006; Ghori et al., 2016) that has also been certified Generally Recognized As Safe (GRAS) by the Food and Drugs Administration (FDA). By incorporating it into the scaffold we achieved controlled and localized delivery, to the injured area, thereby enhancing the effectiveness of the treatment (Zhang et al., 2019). In the realm of SCI, scaffolds serve as a structural foundation, facilitating the growth and regeneration of compromised neural tissue (Koffler et al., 2019; Cheng et al., 2022). A current focus of investigation involves thymol-loaded nanofibrous scaffolds, under scrutiny for their potential to augment tissue regeneration and mitigate pain (Chen et al., 2020; Yue et al., 2021). The underlying principle driving the utilization of thymol-loaded nanofibrous scaffolds revolves around the fusion of thymol’s therapeutic advantages with the supportive attributes inherent to nanofibrous scaffolds.

## 2 Methods and materials

### 2.1 Materials

Poly (vinyl alcohol) (PVA, Mw: 72 kDa), chitosan (CS, MW: 190–300 kDa, 75%–85% deacetylated), 2,2-diphenyl-1-picrylhydrazyl (DPPH) and thymol (CAS No: 89-83-8) were obtained from Sigma-Aldrich (Steinheim, Germany). Acetic acid and ethanol were bought from Merck Company (Darmstadt, Germany).

### 2.2 Fabrication of thymol-loaded nanofibrous scaffolds

The blank PVA/CS nanofibrous scaffold and the scaffold containing thymol were fabricated with the electrospinning technique. To prepare the blank scaffold, a mixture of PVA (10% w/v) and CS (2% w/v) was blended at the ratio of 80:20 v/v. Then, the polymeric mixture was subjected to electrospinning at room temperature (25°C), set at +20 kV applied voltage, flow rate 1 mL/h, and a 15 cm tip-to-collector distance. Likewise, thymol (5–15 wt%) was added to the PVA/CS blended solution and was converted to the electrospun fibers at the same condition. After that, they were removed from the aluminium sheet and stored for further characterizations and examinations.

### 2.3 Characterization of the scaffolds

Scanning electron microscopy (S-4700, Hitachi Ltd., Tokyo, Japan) at an accelerating voltage of 30.0 kV after a gold plasma sputtering characterized the scaffolds’ morphology and diameter distribution. Besides, studying the interactions between the scaffold ingredients and endorsing the incorporation of thymol was conducted using Fourier transform infrared (FT-IR) spectrophotometer (IR Affinity 1S, Shimadzu, Japan) from 400 to 4,000 cm^−1^ at room temperature. The water absorption capacity and the surface wettability of the scaffolds were determined by immersion in water (pH 7.4) at 37°C and water contact angle measurement through the sessile drop technique, respectively (Doostan et al., 2023). Moreover, the antioxidant activity of the prepared scaffolds was performed via DPPH radical scavenging assay as previously described (Mastelić et al., 2008).

Furthermore, thermal analysis was conducted using Differential Scanning Calorimetry (DSC) to elucidate the thymol-polymer interactions and structural transitions (Zeugolis and Raghunath, 2010). The samples, including pure thymol, blank scaffold, and loaded scaffold, were analyzed with a thermal analyzer (NETZSCH STA 449 F3 Jupiter, Germany) under a nitrogen atmosphere from 25°C to 100°C at a rate of 10°C/min.

### 2.4 *In vivo* model for spinal cord injury

In the study, 42 adult male Wistar rats weighing (220–260 g) were bought from Aftab Lorestan and held in the central animal house, at Kermanshah University of Medical Sciences. Animals were fed *ad libitum* and kept under standard conditions (light/dark cycle for 12 h in relative humidity of 60% ± 5% and temperature of 24°C ± 2°C). Procedures were performed following the policies for the treatment and care of laboratory animals published by The Iranian National Institute of Health and approved by the Ethics Committee at the Kermanshah University of Medical Sciences (Ethical code: IR.NIMAD.REC.1400.108). All actions were made to relieve mouse distress.

Rats were randomly split into seven groups each six rats as follows: 1) Group 1 (Sham group): received laminectomy without injury and were treated with normal saline; 2) Group 2: received compression injury; 3) Group 3: received compression injury and were treated with thymol (5 mg/kg, intraperitoneally); 4) Group 4: received compression injury and were treated with just the blank scaffold (*in-situ*); 5) Group 5: received compression injury and were treated with the nanofibrous scaffold containing 5 wt% thymol (*in-situ*); 6) Group 6: received compression injury and were treated with the nanofibrous scaffold containing 10 wt% thymol (*in-situ*); 7) Group 7: received compression injury and were treated with the nanofibrous scaffold containing 15 wt% thymol (*in-situ*).

### 2.5 Induction of spinal cord injury via compression

Animals were transferred to the laboratory before testing to be adapted to the environment. Anesthesia was induced in rats, by the mixture of ketamine (80 mg/kg) and xylazine (10 mg/kg) injected intraperitoneal (IP). The surgical area was shaved and disinfected with ethanol 70%. Laminectomy was accomplished at the T8-T9 level with a micro rongeur (Fine Science Tools, United States). Henceforward, the extradural clip compression SCI caused damage by closing an aneurysm clip (Aesculap, Tuttlingen, Germany) with a 90 g calibrated closing force, near the spinal cord for 1 min (Poon et al., 2007; Cheriyan et al., 2014; Bagheri Bavandpouri et al., 2024). Then muscles and skin were sutured, and rats were permitted to recover on a 30°C heating pad, and then received saline (2 mL, twice a day, subcutaneously) and cefazoline (40 mg/kg twice on the day of surgery, i.p.) to rehydrate and control urinary tract infections. SCI rendered all animals thoroughly paraplegic. Urinary excretion was manually dragged out two times per day until achieving bladder-emptying reflex comeback.

### 2.6 Weight change measurement

The rats were weighed at the commencement of experiments and repeated on days 7, 14, 21, and 28. The weight changes in each group were calculated through the directions below:

Weight difference = (Animal’s weight on days 7, 14, 21, and 28 – weight on day 0 (before the surgery)).

### 2.7 Functional recovery assessment using Basso, Beattie, and Bresnahan test

Basso, Beattie, and Bresnahan (BBB) is a locomotor behavior test that is a proper and predictive estimation of locomotor recovery and can also differentiate behavioral developments due to separate injuries (Basso et al., 1995). The approach was based on evaluated behavior before the injury, on the first postoperative day, and then for up to 4 weeks on days 1, 7, 14, 21, and 28. Scoring categories were determined and classified based on the experimental ordering of the Basso, Beattie, and Bresnahan Locomotor Rating Scale.

### 2.8 Mechanical sensory testing with von-Frey Filaments

The von Frey test is a gold standard for specifying mechanical thresholds, which were invented by Maximilian von Frey and evaluating mechanical allodynia in mice and rats (Deuis et al., 2017). In this test, a monofilament was used vertically to the plantar surface of the rat’s rear paw until its apex, producing a stable pre-determined force (typically 15–180 g for rats) for 2–5 s. When any quick paw withdrawal, licking, or shaking was observed while applying the stimulus, it was considered a positive response. The test was repeated on days 7, 14, 21, and 28.

### 2.9 Assessing sensory response with acetone drop test

Cold chemical and thermal sensitiveness were estimated by utilizing the acetone drop method (Yoon et al., 1994; Ruan et al., 2018). The technique involved applying (100 μL) of acetone using a micropipette to the rat’s hind paw through the wired mesh cage. The rats were placed in the cage and allowed to acclimate for approximately 20 min before acetone application to facilitate exploration of their surroundings. The acetone drop was applied mildly to the rat’s rear paws. Rats’ reactions such as paw licking, shaking, or rubbing the hind paw and brisk foot withdrawal typically 2–5 s after the acetone administration were documented as positive responses. For each measurement, the paw was sampled three times with an interval of 5 min calculated, which scored 0, 1, 2, 3 and 4, respectively. The test was repeated on days 7, 14, 21, and 28.

### 2.10 Thermal sensitivity assessment using hot plate test

The hot-plate test scored behavioural for measurements to estimate acute pain sensitivity (Hunskaar et al., 1986). Rats were put, each one separately, on a surface maintained at 50°C–56°C. The rat’s movement was restricted by a plexiglass cylinder. The latency to hind paw licking, shaking, or jumping, was recorded by time, and at this point, the animal was instantly released from the hot plate to lessen the suffering. The test was repeated on days 7, 14, 21, and 28.

### 2.11 Histopathological evaluation of spinal cord injury

All rats were sacrificed (100 mg/kg ketamine + xylazine 20 mg/kg) at the end of day 28 when the behavioral tests were finished. The spinal cord tissue was attentively extracted and then kept in 200 mL of 4% paraformaldehyde and 0.1 M phosphate-buffered saline (PBS). The tissues were then processed (Didban sabz DS2080/H; Iran). The sections of the spinal cord in each rat were stained with H&E. Histopathological alterations were analyzed using a light microscope, and the data was collected at a magnification of 40X.

### 2.12 Statistical analysis

Statistical analyses were performed using GraphPad Prism software version 6. Results are presented as mean ± standard error of the mean (SEM). One-way or two-way analysis of variance (ANOVA) was conducted, followed by Tukey’s or Bonferroni *post hoc* tests, to determine significant differences between groups. A *p*-value of less than 0.05 was considered statistically significant (Fakhri et al., 2018; Bagheri Bavandpouri et al., 2024).

**FIGURE 1:**
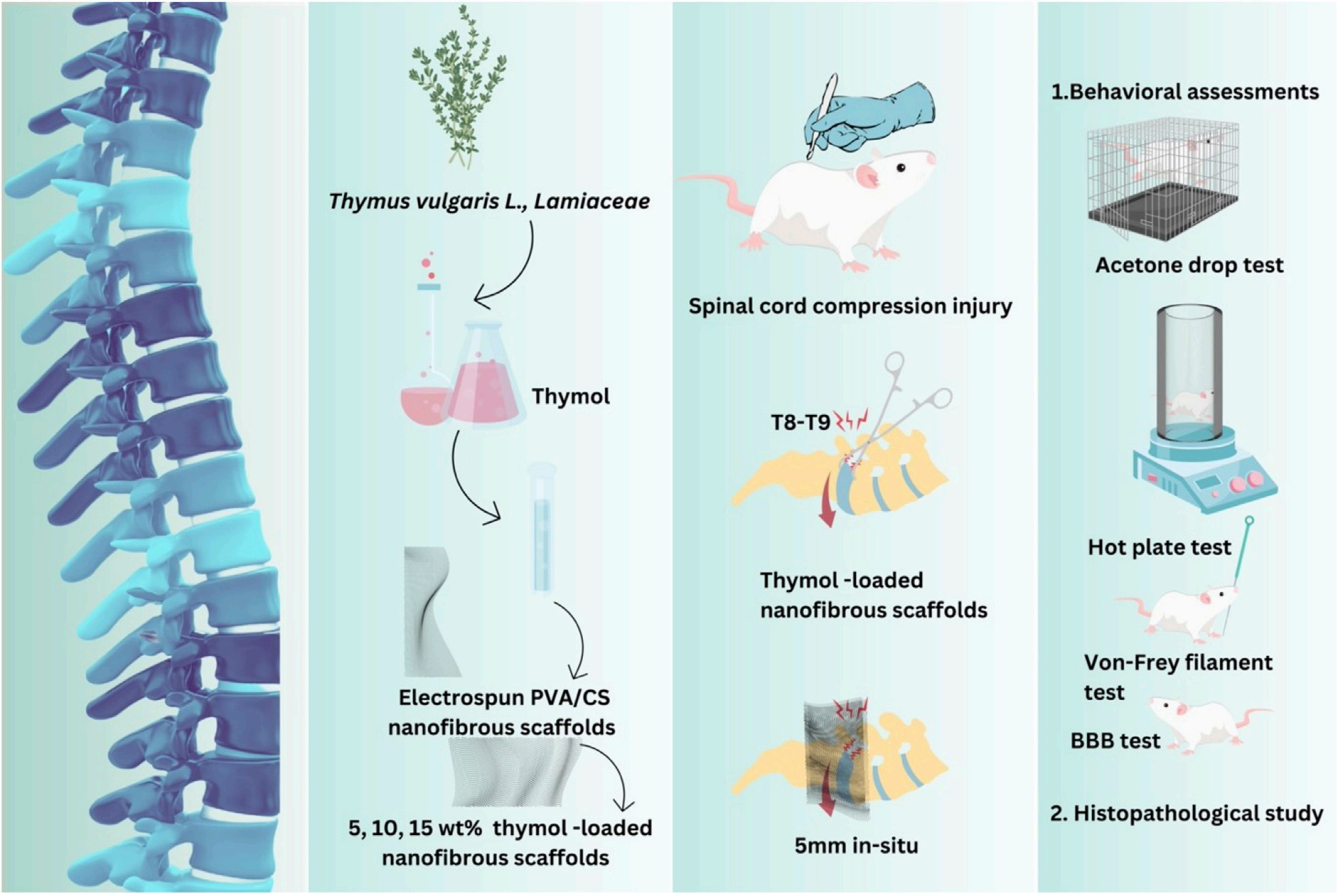
Experimental design was conducted using a rat compression model of SCI to evaluate the therapeutic potential of poly(vinyl alcohol)/chitosan (PVA/CS) scaffolds loaded with varying concentrations of thymol (5, 10, and 15 wt%). The timeline of the experiment included scaffold implantation immediately post-injury, followed by behavioral assessments (e.g., locomotor and sensory function) at regular intervals. Histopathological analyses were performed at the endpoint to evaluate overall tissue regeneration. The design aimed to compare the efficacy of thymol-loaded scaffolds against controls in promoting functional recovery and reducing neuropathic pain associated with SCI.

The experimental design is schematized in Figure 1.

## 3 Results

### 3.1 Morphological characterization of the scaffolds

**FIGURE 2:**
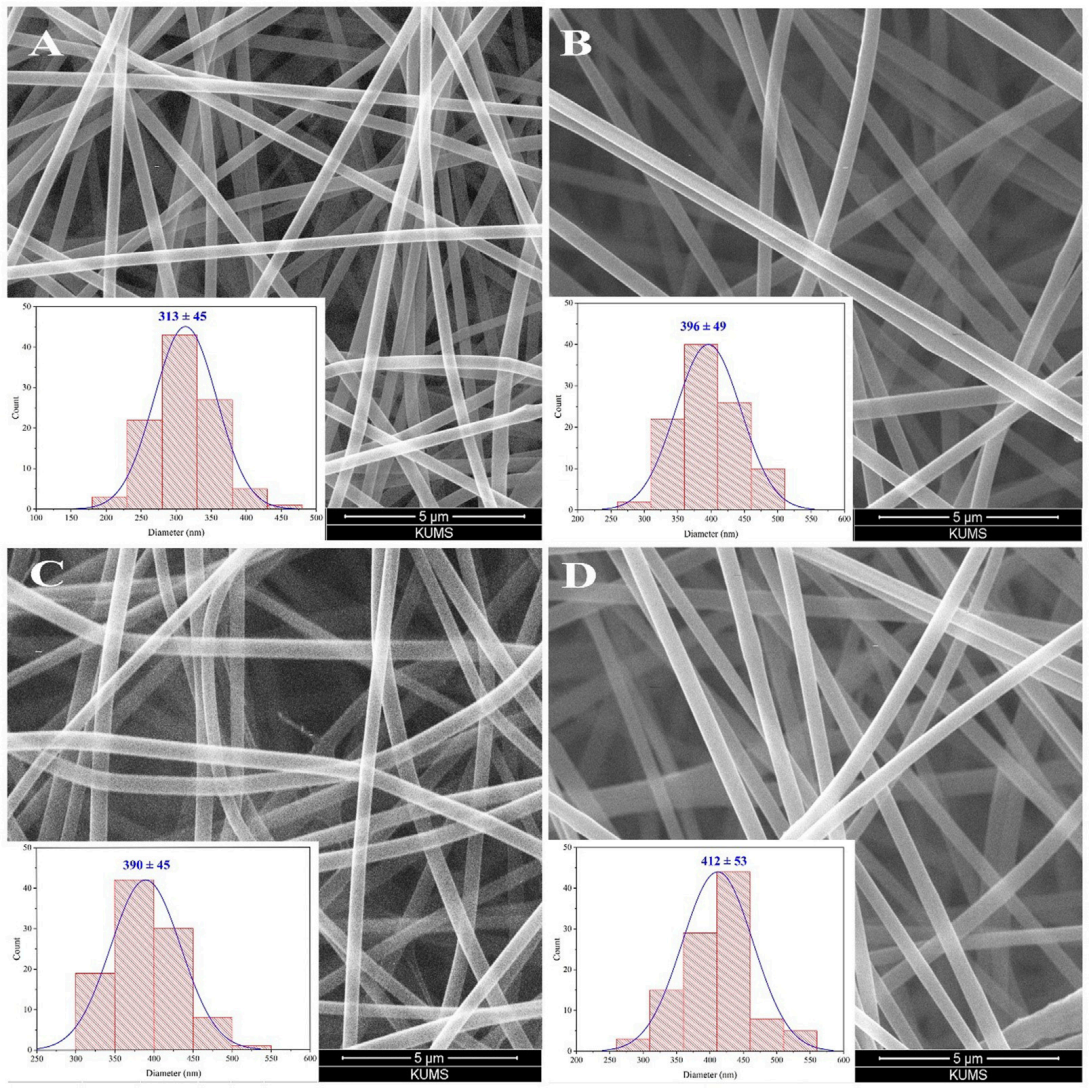
SEM image of blank PVA/CS scaffold **(A)** and the scaffold containing 5 wt% **(B)**, 10 wt% **(C)**, and 15 wt% **(D)** thymol with corresponding diameter distribution histograms.

The SEM micrographs of blank electrospun PVA/CS scaffold and PVA/CS scaffolds containing 5-15 wt% thymol are depicted in Figure 2. The blank scaffold (without thymol) indicated the diameter distribution 313 ± 45 nm and the uniform morphological structure with the smooth, cylindrical, ribbon-shaped, and bead-free NFs with high porous structure (Figure 2A). Adding 5 wt% thymol in the PVA/CS mixture led to increasing the diameter of NFs to 390 ± 45 nm without any change in the morphology (Figure 2B). Likewise, the uniform and beadless NFs with a diameter distribution of 396 ± 49 nm were formed while the thymol concentration was augmented to 10 wt% (Figure 2C). Whereas, adding 15 wt% thymol resulted in the elevation of the average diameter to 412 ± 53 nm with appropriate morphology (Figure 2D). Further incorporation of thymol (>15 wt%) led to the formation of non-uniform NFs in diameter and morphology that is not desirable and was not used.

### 3.2 Characterization of scaffold composition via FT-IR

**FIGURE 3:**
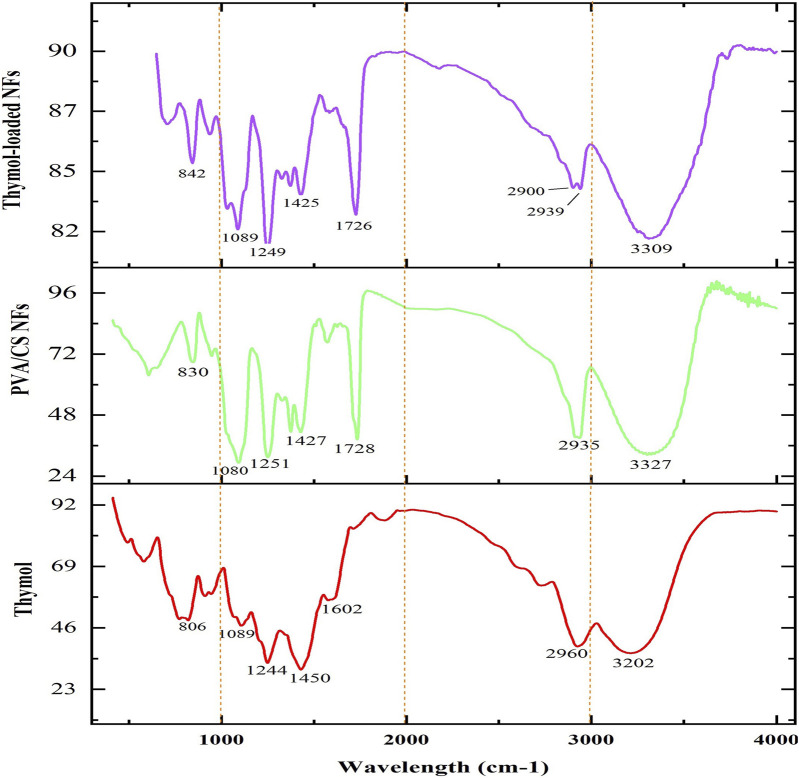
FT-IR spectrum of thymol, blank PVA/CS scaffold, and thymol-loaded PVA/CS scaffold.

FT-IR spectroscopy was performed to verify the chemical structure of samples and identify the presence of thymol and its interaction with the polymeric matrix. The FT-IR spectra of pure thymol, the unloaded (blank), and the thymol-loaded scaffolds are presented in Figure 3. The spectrum of thymol indicated the distinguishing peak at 806 cm^−1^ corresponding to out-of-plane C–H wagging vibrations, and peaks at 1,089 cm^−1^, 1,244 cm^−1^, 1,450 cm^−1^, and 1,602 cm^−1^ are attributed to C–O, C–C and C=C stretching vibrations of the phenolic ring of thymol (Wu et al., 2021). In addition, the characteristic peaks occurring at 2,960 cm^−1^ and 3,202 cm^−1^ are assigned to methyl groups (–CH_3_) and hydroxyl groups (–OH) of thymol, respectively (Sanchez-Rexach et al., 2016; Michalska-Sionkowska et al., 2017). Besides, the spectrum of blank PVA/CS nanofibrous scaffold exhibited characteristic peaks at 1,080 cm^−1^ and 1,251 cm^−1^ that are associated with C–O–C stretching of ether groups as well as a sharp peak at 1728 cm^−1^ corresponding to the C=O stretching bond of the residual acetyl groups in the CS (Rukmani and Sundrarajan, 2012). Also, the absorption peak at 2,935 cm^−1^ is related to the–CH_2_ group, and the broad peak at around 3,327 cm^−1^ is related to the–OH/–NH_2_ vibrations of PVA and the primary amines of CS (Rukmani and Sundrarajan, 2012). Furthermore, the spectrum of thymol-loaded PVA/CS NFs elucidated the prominent peaks similar to both thymol and PVA/CS NFs, confirming thymol’s presence in the NF structure without any adverse interaction as well as increasing intensity of peaks explicates the efficient loading into the nanofibrous scaffold.

### 3.3 Contact angle and water absorption capacity

**FIGURE 4:**
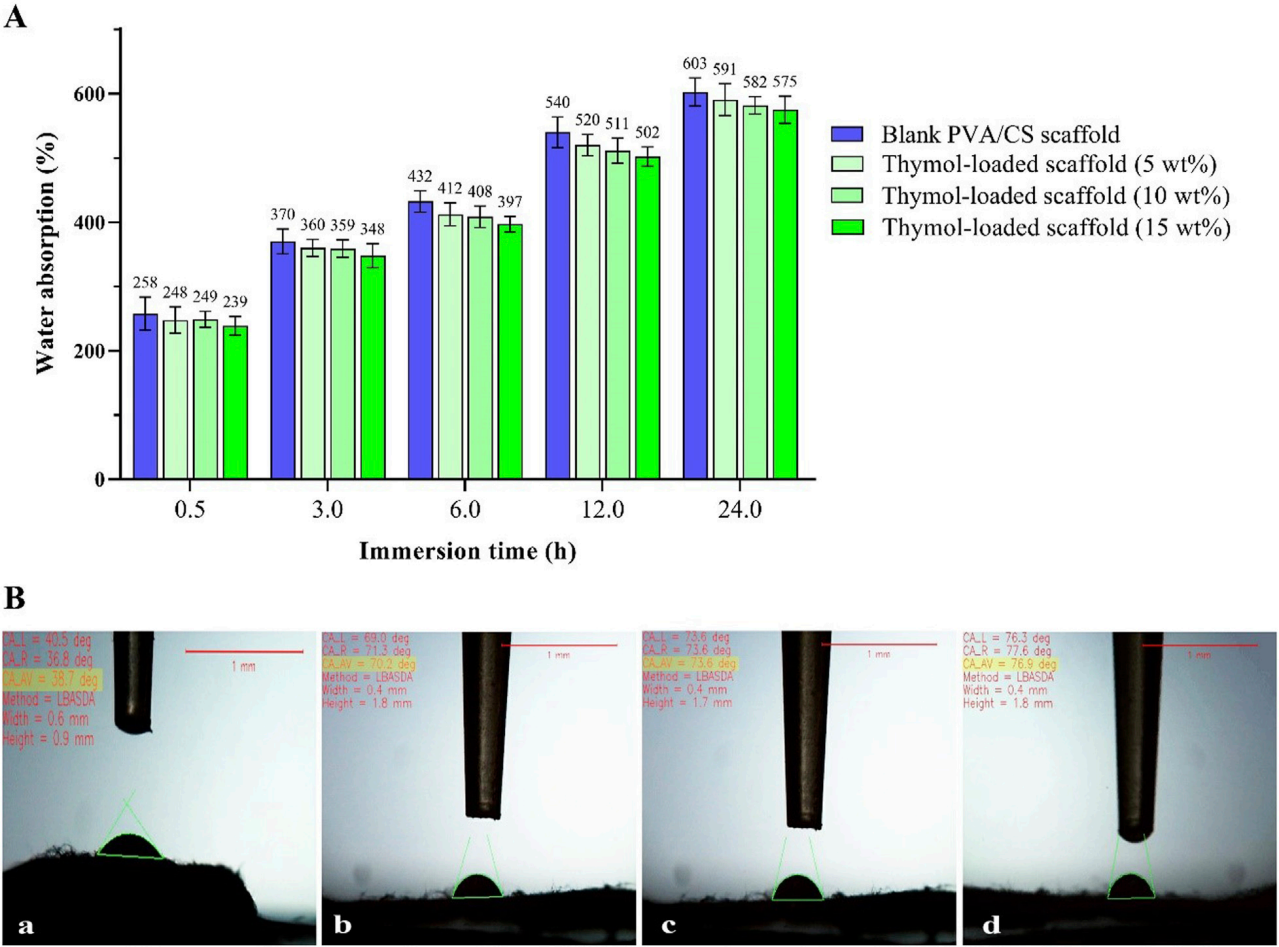
**(A)** The water absorption capacity of the scaffolds at different immersion times, **(B)** The resultant images of the water contact angle of the scaffolds: blank PVA/CS scaffold (a) and the scaffolds containing 5 wt% (b), 10 wt% (c), and 15 wt% (d) thymol with the quantitative values.

The water absorption capability of the prepared scaffolds is depicted in Figure 4A. In the 30-min immersion, the water holding capacity of the blank PVA/CS scaffold was about 258% and reached 603% after 24 h immersion. The thymol-loaded scaffolds (5, 10, and 15 wt%) were able to absorb nearly 248%, 249%, and 239% reached 591%, 282%, and 575% after 24 h immersion, respectively. After 24 h, no change in the amounts of absorption was observed. These findings signify the high and rapid water absorption ability of the scaffolds, which is probably related to their high porosity. Further, the incorporation of thymol did not significantly alter the PVA/CS scaffold’s absorption capability, and there was no significant difference between the scaffolds.

Furthermore, the surface wettability of the scaffolds was evaluated through the water contact angle shown in Figure 4B. The measured contact angle of the blank PVA/CS scaffold was about 38.7°. While these values were 70.2°, 73.6°, and 76.9° for the loaded scaffolds containing 5, 10, and 15 wt% thymol, respectively. These results indicate the prepared scaffolds have a highly hydrophilic and wettable surface, affecting biological response, protein adsorption, and cellular attachment (Doostan et al., 2023).

### 3.4 Thermal properties of scaffold samples via DSC

**FIGURE 5:**
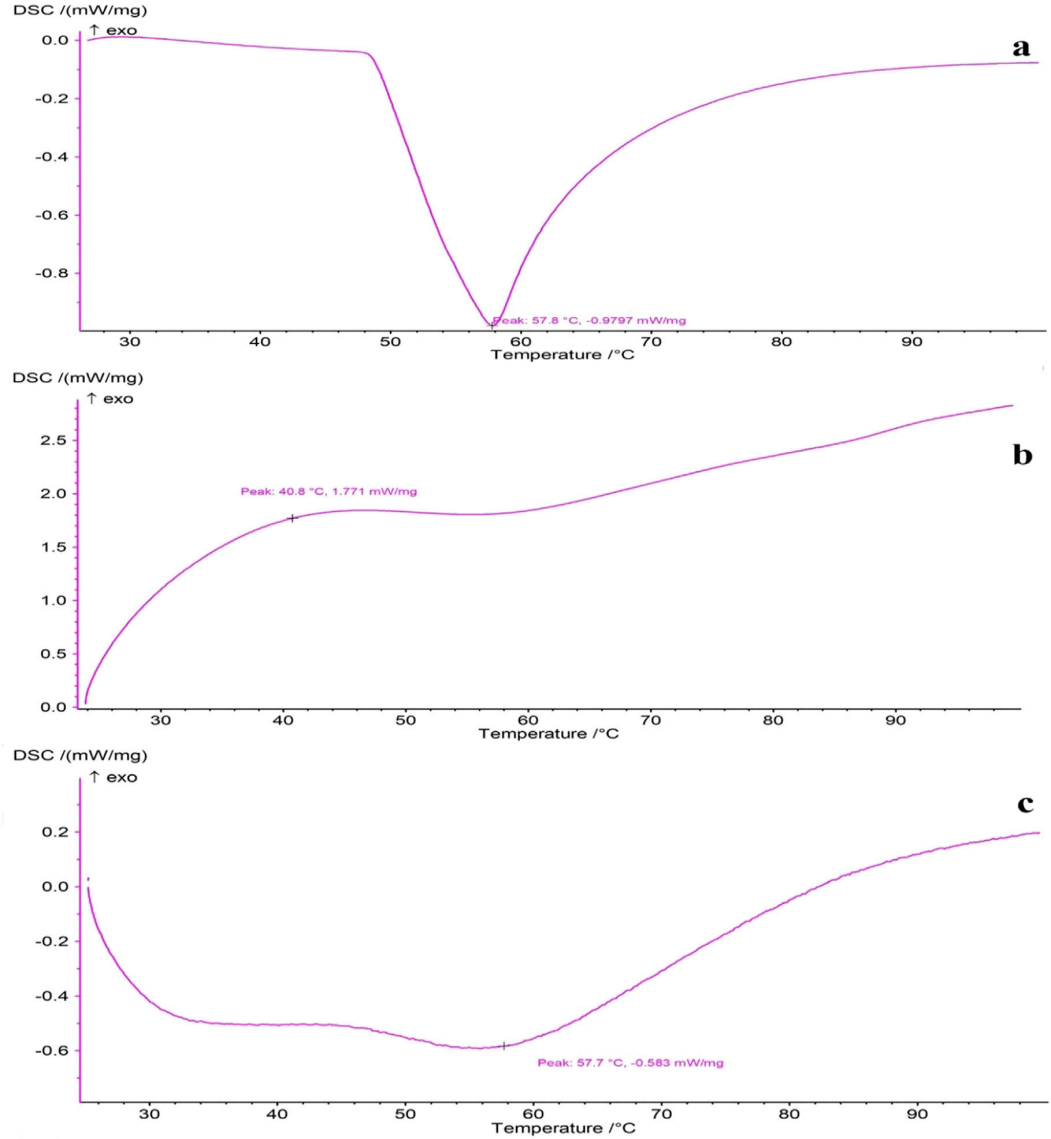
DSC thermograms of **(A)** pure thymol, **(B)** blank PVA/CS scaffold, and **(C)** thymol-loaded scaffold.

The DSC thermograms of pure thymol and the prepared scaffolds are displayed in Figure 5. In the DSC curve of thymol, a sharp endothermic peak appeared around 57°C, corresponding closely to its melting point (Zamani et al., 2015; Giotopoulou et al., 2024). The blank PVA/CS scaffold did not exhibit sharp peaks, indicating the absence of crystalline microstructure. The majority of polymeric chains in the non-crystalline state in the electrospun NFs is likely due to the rapid solidification of stretched polymeric chains during the electrospinning process (Jia et al., 2007; Gholipour, K et al., 2009; Olvera Bernal et al., 2023). Likewise, the DSC thermograph of the thymol-loaded scaffold indicates that the characteristic melting endothermic peak of thymol disappeared upon incorporation into the electrospun NFs. This result implies that thymol converts from crystalline to amorphous form, presumably due to rapid solvent evaporation and restricted molecular mobility caused by intermolecular interactions, e.g., hydrogen bonds, during electrospinning (Chen et al., 2020; Jiffrin et al., 2022). Such conversion, leading to the molecular dispersion of thymol during incorporation into nanofibrous scaffolds, can provide homogeneous distribution and higher drug loading in the nanofiber matrix, ensuring consistent release profiles, which is advantageous for poorly soluble drugs, enhancing solubility, bioavailability, and dissolution rate (Qi and Craig, 2016; Jiffrin et al., 2022).

### 3.5 DPPH radical scavenging assay

**FIGURE 6:**
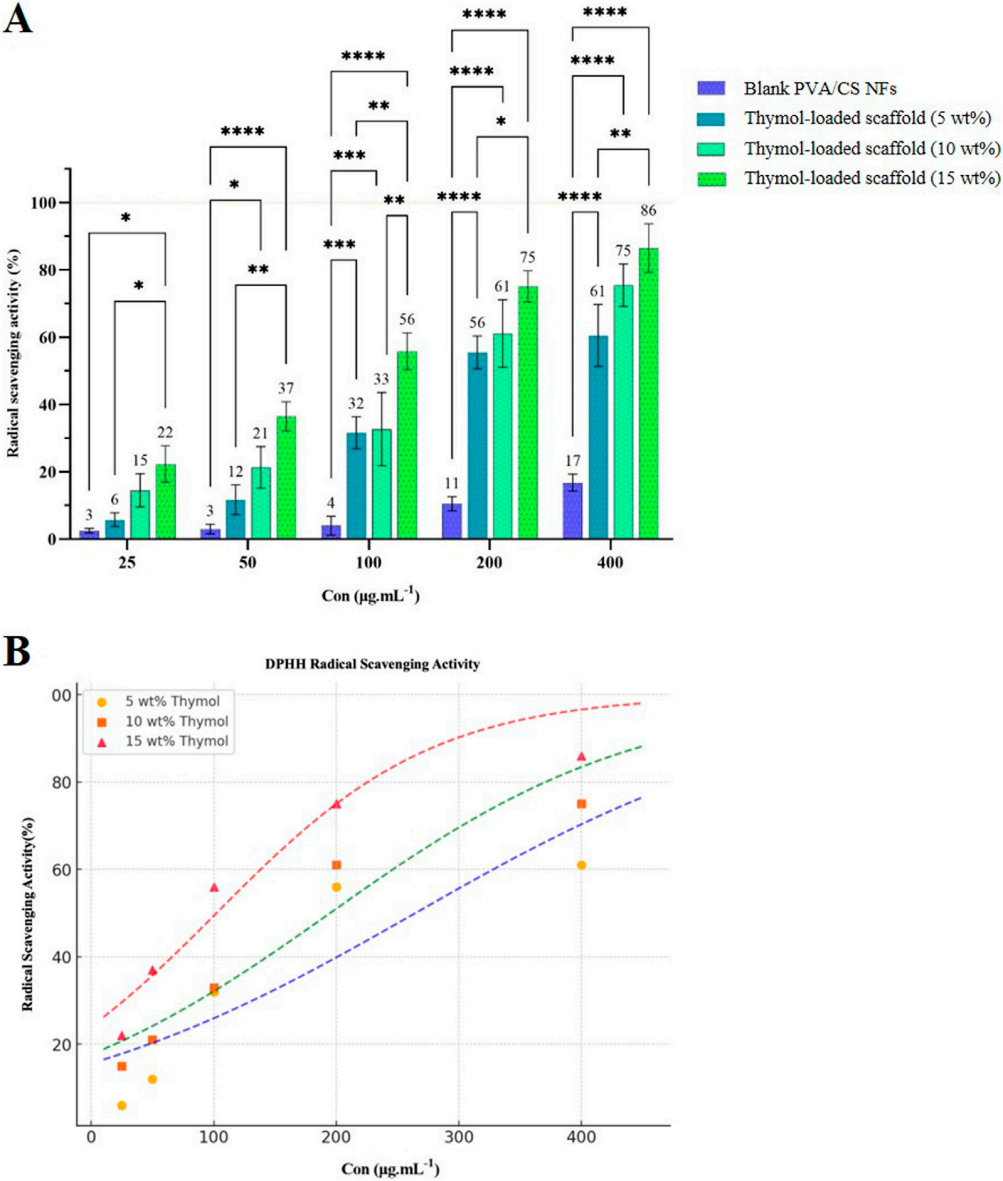
**(A)** Free radical scavenging activity of thymol-loaded and blank PVA/CS scaffolds at different concentrations. **(B)** Determination of IC_50_ values and sigmoidal curve fitting for representing radical scavenging activity of the scaffolds.

The antioxidant activity of the scaffolds was determined by measuring the DPPH free radical scavenging strength; the results of which are shown in Figure 6A. The blank scaffold showed low antioxidant activity (17%) at high concentrations, possibly owing to the absorption of DDPH molecules by the large surface area of the NFs. The antioxidant activity of the scaffolds was augmented significantly by adding thymol. The activities of the scaffolds rose significantly by adding thymol and further increased in higher quantities. At concentrations of 25–400 μg/mL, the scavenging activities of the scaffolds containing 5, 10, and 15 wt% thymol were found to be from 6%–61%, 15%–75%, and 22%–86%, respectively. The higher loading of thymol resulted in higher antioxidant activity that is due to the presence of thymol in the scaffolds, enabling to donate of hydrogen by hydroxyl groups in the structure and breaking the chain reactions of free radicals (Aziz and Almasi, 2018). Scaffolds exhibited a concentration-dependent activity for radical scavenging, and a rise in thymol content increased antioxidant activity. Blank nanofibrous scaffold of PVA/CS exhibited no radical scavenging activity, but 5, 10, and 15 wt% thymol-loaded scaffolds exhibited significantly increased antioxidant activity at all concentrations (*p* < 0.05). To further evaluate the antioxidant efficacy, a half-maximal inhibitory concentration (IC_50_) value was determined with a sigmoidal fitting model Figure 6B. The IC_50_ values, representing a 50% free radical-scavenging activity, were calculated as: 5 wt% thymol-loaded scaffold: 264.52 µg, 10 wt% thymol-loaded scaffold: 194.93 µg, 15 wt% thymol-loaded scaffold: 102.14 µg. These results confirm that the 15 wt% thymol-loaded scaffold exhibited the highest antioxidant activity, with its lowest IC_50_ values. Dose-dependent enhancement in free radical-scavenging activity confirms that thymol is a key player in contributing towards the antioxidant activity of nanofibrous scaffolds, and it can contribute towards minimizing oxidative stress in SCI.

### 3.6 Weight change measurement

**FIGURE 7:**
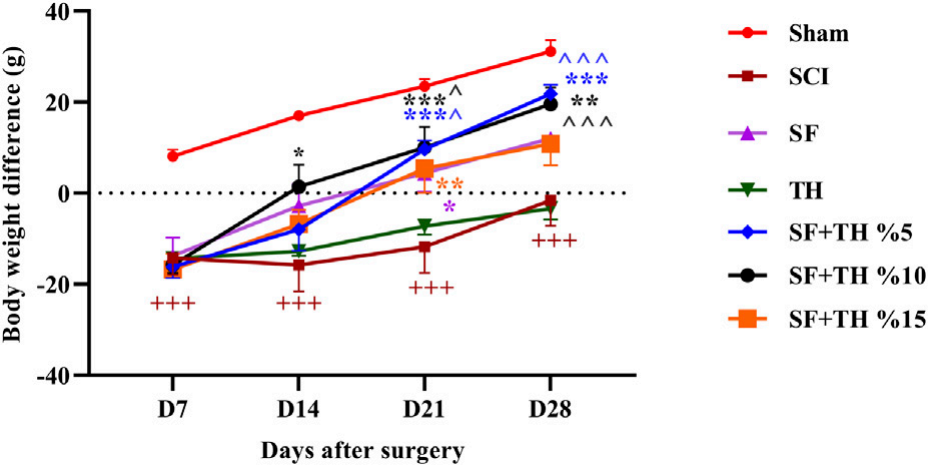
Investigating the effect of PVA/CS scaffold loaded with thymol on weight changes following SCI in rats. Data are presented as mean ± SEM (*n* = 6). ( ^*p* < 0.05 and ^ ^ ^*p* < 0.001) vs. TH group, ***p < 0.001, **p < 0.01, *p < 0.05) vs. SCI group and (+++ *p* < 0.001) vs. Sham group.

The weight changes of the animals compared to the day before surgery were evaluated and are shown in Figure 7. The obtained results indicate that the sham group showed a normal weight gain pattern over the 4-week period. However, in the injured rats compared to the Sham group, a significant impairment in weight gain was observed during the weeks following the injury (*p* < 0.001). Treatment with thymol-loaded scaffolds at 10% and 5% resulted in a significant increase in the normal weight of the animals during the 21 days after the injury (*p* < 0.001), while thymol-loaded scaffolds at 15% led to a notable increase as well (*p* < 0.01) on day 21. This observed improvement eventually compensated for the weight loss caused by the injury, and no significant differences were observed between the treatment groups.

### 3.7 Basso, Beattie, and Bresnahan test

**FIGURE 8:**
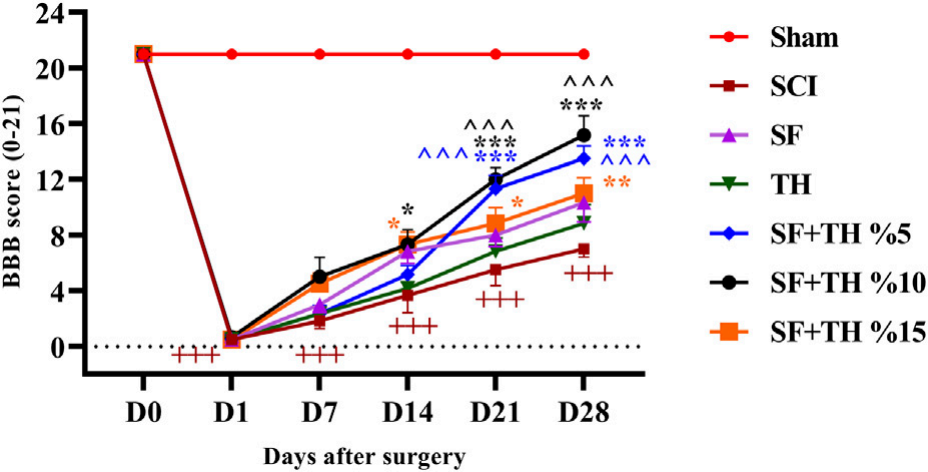
Investigating the effect of PVA/CS scaffold loaded with thymol on motor performance in the BBB test following SCI in rats. Data are presented as mean ± SEM (*n* = 6). ( ^ ^ ^*p* < 0.001) vs. TH group, (***p < 0.001, **p < 0.01, *p < 0.05) vs. SCI group and (+++*p* < 0.001) vs Sham group (SF: blank PVA/CS scaffold, TH: free thymol, SF + TH: scaffolds containing thymol).

Motor performance of rats after SCI was measured by the BBB scale as shown in Figure 8. Examining the movement performance of the sham group indicated no movement disorders after laminectomy, and the rats consistently scored 21 on all examined days. In contrast, the SCI group exhibited complete paralysis on the first day after the injury, with scores of 0 and 1, and throughout the treatment period, they showed a significant decrease in motor performance compared to the sham group animals (*p* < 0.001). However, the groups treated with 10% and 15% thymol-loaded scaffolds showed an increase in the recovery of movements and joints by day 14 after the injury (*p* < 0.05), and there was a significant improvement in motor performance for the group treated with 5% and 10% thymol-loaded scaffolds on days 21 and 28 (*p* < 0.001).

### 3.8 Von-Frey filament test

**FIGURE 9:**
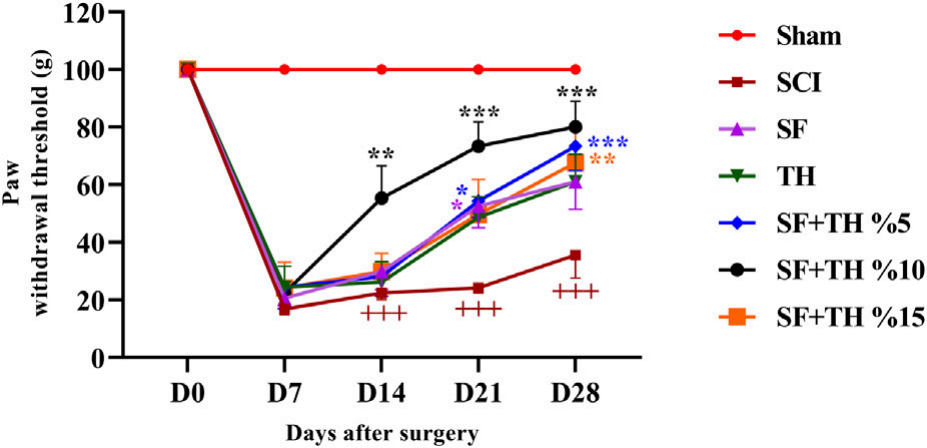
Investigating the effect of PVA/CS scaffold loaded with thymol on mechanical pain tolerance threshold following SCI in rats. Data are presented as mean ± SEM (*n* = 6). ***p < 0.001, **p < 0.01, *p < 0.05) vs. SCI group and (+++ *p* < 0.001) vs. Sham group (SF: blank PVA/CS scaffold, TH: free thymol, SF + TH: scaffolds containing thymol).

Evaluation of mechanical pain was performed by applying the mechanical force of von Frey fibers on the dorsal surface of the rat’s hind legs, between the 2^nd^ and 3^rd^ toes (Figure 9). The obtained results show that in the sham group, the mechanical pain tolerance threshold remained consistent during the treatment period after laminectomy. On the other hand, in the SCI group, a significant decrease in the mechanical pain tolerance threshold was observed on day 7, and no significant changes were observed in the following days compared to the sham group (*p* < 0.001). The group treated with a 10% thymol-loaded scaffold demonstrated an improvement, showing a more pronounced reduction in neuropathic pain caused by foot injury on day 14 (*p* < 0.01), and continued to exhibit reduced pain levels on days 14, 21, and 28 (*p* < 0.001). Additionally, the group treated with 5% thymol-loaded scaffolds showed a reduction in neuropathic pain on day 7 (*p* < 0.05) and sustained the reduction on day 28 (*p* < 0.001).

### 3.9 Acetone drop test

**FIGURE 10:**
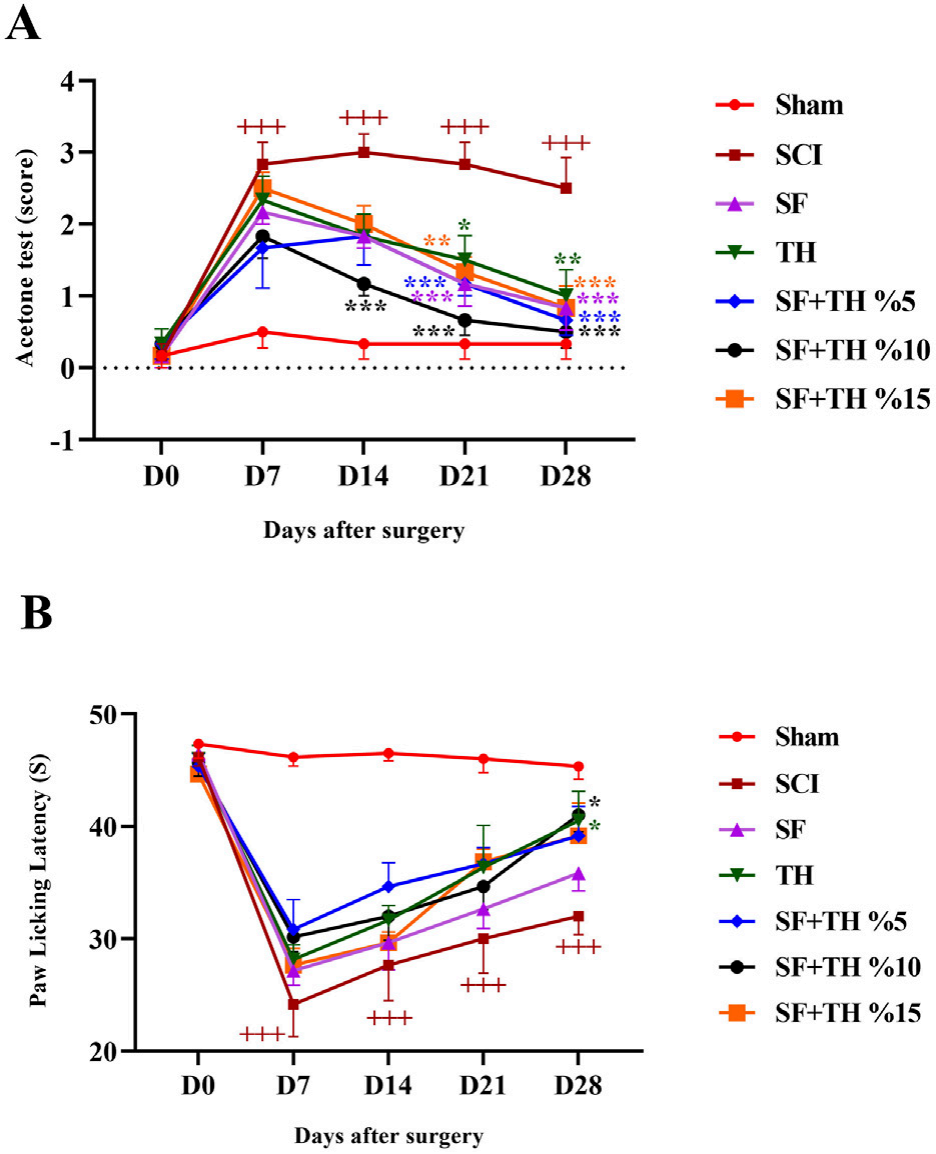
Investigating the effect of PVA/CS scaffold loaded with thymol on cold **(A)** and thermal **(B)** pain tolerance threshold following SCI in rats. Data are presented as mean ± SEM (*n* = 6). (***p < 0.001, **p < 0.01, *p < 0.05) vs SCI group and (+++ *p* < 0.001) vs Sham group. (SF: blank PVA/CS scaffold, TH: free thymol, SF + TH: scaffolds containing thymol).

The acetone drop test was used to assess the animals’ tolerance threshold to the cold pain stimulus and the data are depicted in Figure 10A. In the sham group, the foot withdrawal reflex in response to the cold stimulus (acetone) remained consistent, and no significant changes were observed in the days following laminectomy. This is despite the fact that the injured animals became highly sensitive to cold and their pain tolerance threshold decreased more clearly, also there is a significant difference in the comparison of the reflexes of this group with the Sham group (*p* < 0.001). Treatment with a 10% thymol-loaded scaffold increased the tolerance of the response threshold to cold stimulus from day 14 until day 28 after injury (*p* < 0.001) and also 5% thymol-loaded scaffold and blank scaffold increased the tolerance of the response threshold to the cold stimulus at day 21 (*p* < 0.001). In contrast, the 15% thymol-loaded scaffold showed a moderate increase at day 21 (*p* < 0.01).

### 3.10 Hot plate test

Thermal pain was assessed using the hot plate test, as shown in Figure 10B. The obtained results indicated that the time delay in foot licking, as a measure of pain tolerance threshold due to heat in the sham group, did not change significantly from the first to the 28th days after laminectomy. Compared to the sham group, the SCI group showed a much lower tolerance to thermal pain on days 7 and 14 (*p* < 0.001) and in the last 2 weeks, the pain tolerance threshold increased slightly in the direction of recovery (*p* < 0.001). Treatment with a 5 mg/kg dose of thymol and 10% thymol-loaded scaffold was associated with a moderate increase in the tolerance threshold of response to thermal stimuli with a relatively mild slope in the final week after injury (*p* < 0.05).

### 3.11 Histopathology analysis

**FIGURE 11:**
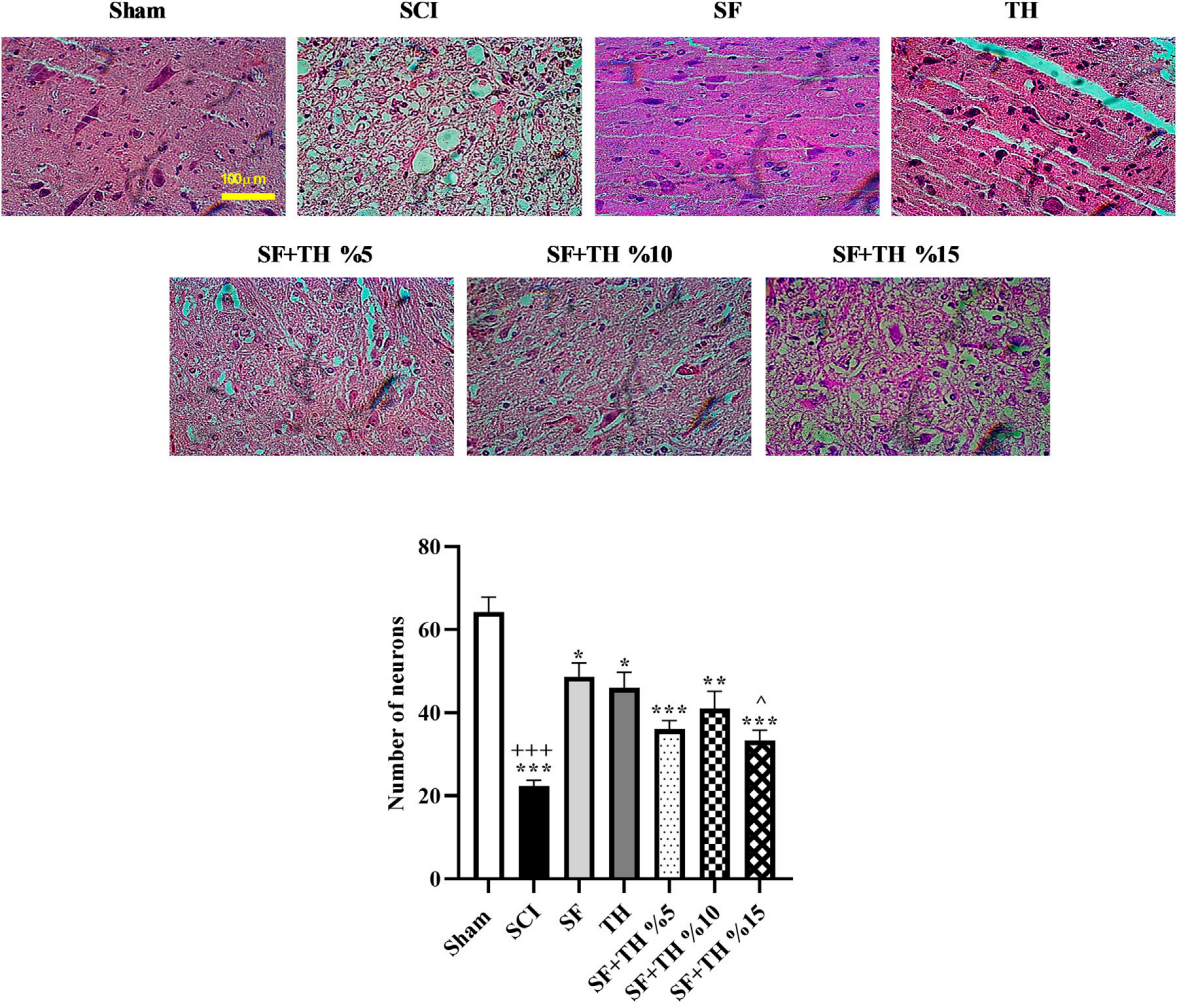
**(A)** Investigating the effect of PVA/CS scaffold loaded with thymol on changes in the number of motor neurons in the ventral horn of the spinal cord in hematoxylin-eosin staining of the transverse sections prepared from the spinal cord with 40x magnification following SCI in rats. **(B)** Data are presented as mean ± SEM (*n* = 3). (^*p* < 0.05) vs. TH group, (***p < 0.001, **p < 0.01, *p < 0.05) vs. SCI group and (+++ *p* < 0.001) vs. Sham group (SF: blank PVA/CS scaffold, TH: free thymol, SF + TH: scaffolds containing thymol).

Hematoxylin-eosin staining was used to investigate the extent of tissue damage after SCI, as depicted in Figure 11A. Counting the number of motor neurons (Figure 11B) in the area of the ventral horn of the gray matter of the spinal cord on day 28 after the injury revealed a significant decrease in the number of neurons at the injury site (*p* < 0.001). Treatment with 10% and 5% thymol-loaded scaffolds demonstrated improved protection of neurons and led to an increase in the number of motor neurons in the ventral region of the spinal cord (*p* < 0.001). Similarly, 15% thymol-loaded scaffold treatment also showed improvement in the number of motor neurons (*p* < 0.01).

## 4 Discussion

SCI is a devastating condition that occurs when the spinal cord suffers damage (McDonald and Sadowsky, 2002). This damage leads to the loss of motor, sensory, and autonomic functions, below the injury site (Anjum et al., 2020). This is a life-altering condition that permanently affects an individual’s quality of life and independence (Norrbrink Budh et al., 2006). Unfortunately, there is currently no cure for SCI, and treatment options are limited. The main focus of managing SCI revolves around preventing damage, stabilizing the spine, and providing care to manage complications (Dietrich, 2015). One notable avenue of inquiry pertains to the utilization of stem cell transplantation as a regenerative approach (Nakamura and Okano, 2013; Tahmasebi and Barati, 2022). Within this domain, extensive research is underway, encompassing various stem cell types, including induced pluripotent stem cells (Trawczynski et al., 2019) as well as adult stem cell variants like neural stem cells (Wang J. et al., 2020) and mesenchymal stem cells (Xu and Yang, 2019). Notably, promising outcomes have been observed in studies examining these cell types. Concurrently, another sphere of exploration centers on the deployment of biomaterials in the capacity of scaffolds to facilitate regeneration (Shen H. et al., 2022). These biomaterials, which encompass hydrogels, nanofiber scaffolds, and conductive polymers, serve the pivotal role of providing structural support while creating a conducive environment that fosters cell adhesion, migration, and differentiation (Zhang et al., 2019).

Scaffolding technology has gained significance in tissue engineering, especially in the treatment of SCI (Zhang et al., 2019). This is achieved by supplying a framework that assists in cell building and tissue repair (Shrestha et al., 2014). Extracellular matrices are often not spared in spinal cord injuries, as they are often damaged. This disruption eliminates the matrix that facilitates cell attachment, movement, and change, all of which are key steps in the restoration of nerves. Such aggravated conditions can be overcome by using nanofibrous scaffolds that can restore ECM-like three-dimensional biological structures for the culture of neural tissue (Ma et al., 2021). Due to its high surface area and porosity, the scaffold allows the important nutrients and oxygen to diffuse into the cells, as well as any signaling molecules required for the cells to survive and the tissue to regenerate. This structure is particularly useful in handling situations of SCI where there is a need to localize delivery of treatment, as there is secondary damage that occurs, and there is a need for restoration of function (Snider et al., 2017).

In addition, to their role as a delivery system for molecules and growth factors, biomaterial scaffolds have demonstrated the ability to enhance regeneration and tissue repair, in animal models when combined with cells or growth factors. In this regard, Babaloo et al. designed a scaffold based on poly ε-caprolactone (PCL)/gelatin and seeded by human endometrial stem cells (hEnSCs) were then transplanted into hemisected SCI rats. The results of the study indicated that combination therapy using the differentiated hEnSC seeded on PCL/gelatin scaffolds has the potential to heal the injured spinal cord and limit secondary damage (Babaloo et al., 2019). Investigation led by Fakhri et al., a comprehensive exploration was undertaken, taking into account the pivotal role of glutamate in excitatory toxicity and central sensitization. The primary objective of this study was to assess the impact of ketamine, recognized as an *N*-methyl-D-aspartate (NMDA) receptor antagonist, on sensory-motor functions within a murine model exhibiting SCI. Their robust dataset illuminated compelling findings, revealing a substantial reduction in mechanical allodynia and cold allodynia from the first week through the post-injury within the ketamine-treated group, as compared to the injury group. Their compelling findings collectively underscore the potential therapeutic efficacy of intrathecal ketamine in mitigating sensory-motor dysfunction within a rodent model afflicted by SCI (Fakhri et al., 2020).

Moreover, thymol, an inherent compound found in botanical sources such as thyme, oregano, and basil, emerges as an enticing candidate for multifaceted applications, owing to its well-documented anti-inflammatory, antioxidant, and analgesic properties (Yanishlieva et al., 1999; Braga et al., 2006; Ghori et al., 2016). Thymol displays its inflammatory effects by inhibiting signaling pathways and pro-inflammatory mediators. It aids in reducing the production of cytokines like interleukin 1 beta (IL-1β), tumor necrosis factor-alpha (TNF-α), and interleukin 6 (IL-6), ultimately modulating the response (Nagoor Meeran et al., 2015). Thymol also serves as an antioxidant by scavenging radicals and preventing stress. Achieving this ensures the protection of cells and tissues from damage caused by ROS, while also encouraging their regeneration (Yanishlieva et al., 1999). Moreover, thymol exhibits characteristics by diminishing cell death fostering neuronal survival, and enhancing neurite outgrowth (Timalsina et al., 2023a; Timalsina et al., 2023b). Furthermore, the combination of thymol with a nanofiber scaffold for treating SCI offers various benefits. The nanofibrous scaffold serves as a three-dimensional framework that closely mimics the extracellular matrix, promoting cell attachment and movement, including cells within the damaged spinal cord. Thus when examining the potential of thymol and the PVA/CS scaffold, for SCI treatment it is evident that their combination holds promise. Thymol plays a vital role in facilitating cell attachment and spreading, encouraging cell infiltration into the scaffold. This effect was observed in the Lavanya et al. study, where a drug delivery vehicle using semi-interpenetrating polymer network (SIPN) hydrogels containing sodium alginate and poly (2-ethyl-2-oxazoline) (SA/Pox) loaded with thymol was transplanted into a rat tibial bone defect model system. The study revealed that the incorporation of thymol into SA/Pox/CS hydrogels enhanced bone regeneration. The data demonstrated sustained and prolonged release of thymol from the hydrogels, which promoted osteoblast differentiation *in vitro* and bone formation *in vivo*, indicating its potential application in bone rejuvenation (Lavanya et al., 2023).

**FIGURE 12:**
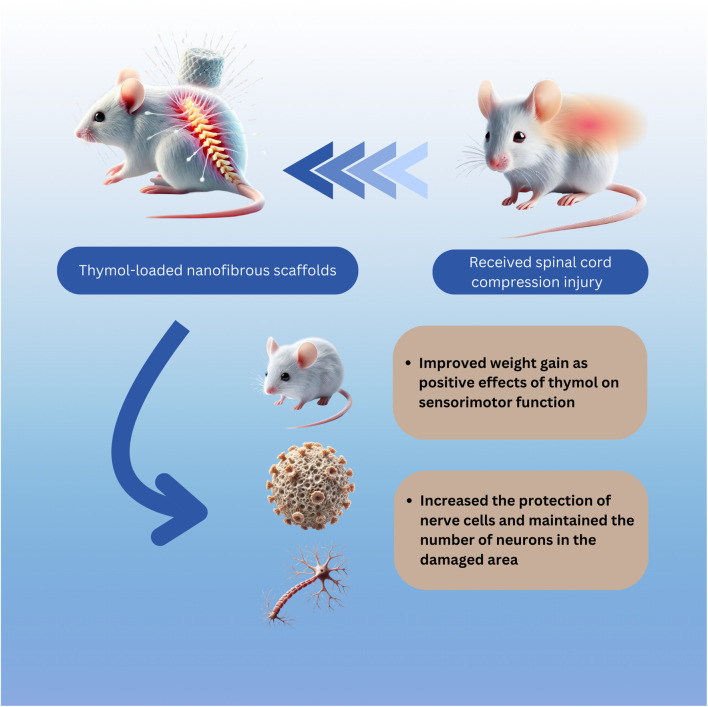
Illustration of rat SCI and being treated with thymol-loaded nanofibrous scaffold. The integration of the thymol-loaded PVA/CS nanofibrous scaffold into the injured spinal cord. The scaffold, characterized by its three-dimensional nanofibrous structure and high porosity, is shown to release thymol at the injury site. Thymol exerts anti-inflammatory, antioxidant, and neuroprotective effects, reducing secondary damage and promoting a regenerative microenvironment. Additionally, the sustained release of thymol enhances functional recovery by alleviating neuropathic pain and improving locomotor and sensory functions.

In the current study, we plan to examine the chemical properties of the PVA/CS scaffold assess its effectiveness through behavioral tests, and confirm the regenerative results by analyzing tissue samples. By merging, the support offered by the scaffold with the advantages of thymol the goal is to enhance functional recovery and stimulate neural regeneration. The focus of this article revolves around an approach that involves combining a nanofiber scaffold with thymol *in-situ* specifically for treating SCI in rats compression model Figure 12, which can accurately control SCI and provide controlled chronic neuropathic pain and movement disorders (Šedý et al., 2008; Cheriyan et al., 2014).

Through this study, significant advancements in the recovery of rats with SCI were observed following treatment with the nanofiber scaffolds containing thymol, as well as improved weight gain. This effect can be possibly attributed to the positive effects of pelargonidin on sensorimotor function, reducing pain, and addressing other disorders related to the injury. The study also investigated the symptoms of neuropathic pain and movement disorders after the compression model of SCI including thermal, cold, and mechanical pain thresholds, as well as the BBB score in rats. The nanofiber scaffolds loaded with thymol, especially at 10 and 5 wt%, demonstrated significant therapeutic effects in reducing motor defects and increasing the pain tolerance threshold. Additionally, the histological study corroborated these findings, showing consistent trends. Counting the number of motor neurons in the ventral horn revealed that the PVA/CS scaffold loaded with thymol significantly increased number of neurons in the damaged area (Figure 12).

## 5 Conclusion

In conclusion, the *in-situ* utilization of PVA/CS nanofiber scaffold containing thymol signifies a breakthrough in the realm of SCI treatment. Fabrication of the scaffolds indicated the formation of NFs with appropriate morphology and uniform size. In addition, the relevant analyses exhibited proper loading of thymol as well as high water absorption, porosity, and wettability properties of the scaffolds. The administration of thymol-loaded scaffolds in animal examinations significantly improved the pain threshold compared to the group with SCI-induced pain. Additionally, the scaffolds restored sensory and locomotor activity. Histopathology studies further revealed enhanced neuronal survival. These findings indicate that the thymol-loaded nanofibrous scaffolds possess neuroprotective and analgesic properties, making them a promising therapeutic intervention for treating neuropathic injuries. This innovative therapy potentially enhances recovery and promotes tissue regeneration among individuals with SCI.

## Data Availability

The raw data supporting the conclusions of this article will be made available by the authors, without undue reservation.

## Abbreviations

ANOVA, analysis of variance; BBB, Basso, Beattie, and Bresnahan; CS, chitosan; DSC: Differential scanning calorimetry; DPPH, (2,2-diphenyl-1-picrylhydrazyl); ECM, extracellular matrix; FT-IR, Fourier transform infrared; GRAS, Generally Recognized As Safe; FDA, Food and Drugs Administration; hEnSCs, human endometrial stem cells; IL-1β, interleukin 1 beta; IL-6, interleukin 6; IP, injected intraperitoneal; NMDA, N-methyl-D-aspartate; NSAIDs, nonsteroidal anti-inflammatory drugs; NFs, nanofibers; PBS, phosphate buffered saline; PCL, poly ε-caprolactone; Pox, poly(2-ethyl-2-oxazoline); PVA, poly(vinyl alcohol); ROS, reactive oxygen species; SCI, spinal cord injury; SEM, Scanning electron microscopy; SIPN, semi-interpenetrating polymer network; TNF-α, tumor necrosis factor-alpha; TRPV1, transient receptor potential cation channel subfamily V member 1.
